# Van der Waals heterostructures of SiC and Janus MSSe (M = Mo, W) monolayers: a first principles study

**DOI:** 10.1039/d0ra04433d

**Published:** 2020-07-07

**Authors:** M. Idrees, M. Fawad, M. Bilal, Y. Saeed, C. Nguyen, Bin Amin

**Affiliations:** Department of Physics, Hazara University Mansehra 21300 Pakistan; Department of Physics, Abbottabad University of Science and Technology Abbottabad 22010 Pakistan binukhn@gmail.com; Institute of Research and Development, Duy Tan University Da Nang 550000 Vietnam nguyenvanchuong2@duytan.edu.vn

## Abstract

Favorable stacking patterns of two models with alternative orders of chalcogen atoms in SiC-MSSe (M = Mo, W) vdW heterostructures are investigated using density functional theory calculations. Both model-I and model-II of the SiC-MSSe (M = Mo, W) vdW heterostructures show type-II band alignment, while the spin orbit coupling effect causes considerable Rashba spin splitting. Furthermore, the plane-average electrostatic potential is also calculated to investigate the potential drops across the heterostructure and work function. The imaginary part of the dielectric function reveals that the first optical transition is dominated by excitons with high absorption in the visible region for both heterostructures. Appropriate band alignments with standard water redox potentials enable the capability of these heterostructures to dissociate water into H^+^/H_2_ and O_2_/H_2_O.

## Abstract 2

Using DFT calculations, we have investigated the electronic structure, Rashba effect, optical and photocatalytic performance of SiC-MSSe (M = Mo, W) van der Waals heterostructures with different stacking patterns of chalcogen atoms.

## Graphical abstract

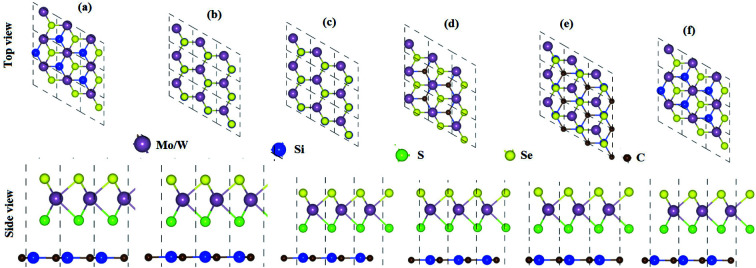

## 1 Introduction

The deposition of Se in MoS_2_ (ref. 1) and S in MoSe_2_ (ref. 2) through chemical vapor deposition (CVD) and correlation with DFT calculations^2^ has led to Janus monolayers with the general formula MXY (M = Mo, W; X, Y = S, Se, Te) emerging as a new class of intriguing materials. An intrinsic electric field due to the breaking of mirror symmetry induces Rashba spin splitting at the Γ-point, making Janus MXY monolayers favorable for two dimensional spintronics.^3^ Induced Rashba spin splitting, vibrational frequency, dipole moment, and band transition in Janus MXY monolayers are found to be associated with differences in atomic radius and electronegativity of chalcogen atoms.^4^ These characteristics also have a significant impact on photocatalysis.^4^

The high recombination ratio of photogenerated electron–hole pairs can hinder the real applications of MXY monolayers as in their parent (MX_2_) phases.^5^ To overcome this issue, layer stacking in the form of van der Waals (vdW) heterostructures with localization of the valence band maximum (VBM) and conduction band minimum (CBM) to two different layers (type-II) is intensively used in designing viable electronic products.^6–14^ The SiC monolayer in a planar geometry is a semiconductor with large in-plane stiffness, high carrier mobility and strong thermal stability. It has a hexagonal lattice and satisfying mismatch with MSSe (M = Mo, W) monolayers (less than 2%), hence the fabrication of SiC-MSSe (M = Mo, W) vdW heterostructures can be realized.^15–19^

SiC-TMDCs vdW heterostructures have type-II band alignment and act as potential photocatalysts for water-splitting at pH = 0.^20^ Recently, direct type-II band alignment and considerable Rashba spin splitting have made GeC-MSSe (M = Mo, W) vdW heterostructures promising candidates for spintronic devices.^21^ Furthermore, appropriate band alignments with standard water redox potentials are predicted to enable the capability of these heterostructures to dissociate water into H^+^/H_2_ and O_2_/H_2_O.

In this work, we perform first principles calculations to investigate the geometry, thermal stability, and electronic properties of SiC-MSSe (M = Mo, W) vdW heterostructures. Furthermore, band alignment (type-I, type-II), Rashba spin splitting, optical and photocatalytic performance of these heterostructures are also investigated.

## 2 Computational details

First principles calculations^22^ are performed in the Vienna *ab initio* simulation package^23–26^ with the Perdew–Burke–Ernzerhof (PBE) functional^27^ and hybrid functional (HSE06).^28^ The long-range dispersion correction method by Grimme^29^ is adopted to describe the weak vdW forces. Plane wave cutoff energy of 500 eV is used for all calculations and a large vacuum of 25 Å is added along the *z* direction of the heterostructures to avoid interactions between the adjacent slabs. We used a *k*-mesh of 6 × 6 × 1 for structural relaxation, and 12 × 12 × 1 for the electronic properties calculations. Geometric relaxation (self consistent iteration) is executed until the force on each atom (energy difference between electronic steps) converges to 0.0001 eV Å^−1^ (10^−5^ eV).

The spin orbit coupling (SOC) effect is incorporated by a second variational method,^30^ in which the scalar relativistic part of the Hamiltonian is diagonalized in a scalar relativistic basis and the calculated eigenfunctions are then used to construct the full Hamiltonian matrix, which can be obtained from: *Ĥψ* = *εΨ* + *Ĥ*_SO_*Ψ*. Furthermore, *ab initio* molecular dynamics simulations are performed to investigate the stability of the heterostructures with an 8 × 8 supercell. The total time and time step are set as 5 × 10^−12^ and 1 × 10^−15^ s, respectively.^31,32^

## 3 Result and discussion

**Fig. 1:**
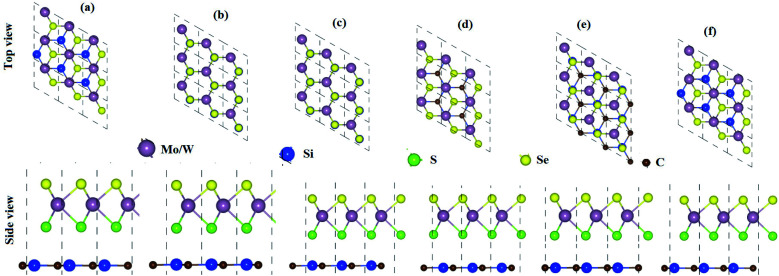
Model-I, SiC-MSSe (M = Mo, W) vdW heterostructures in six possible stacking sequences.

From the optimized lattice constants of MoSSe, WSSe^21^ and SiC^33^ monolayers, vdW heterostructures with twelve favorable stacking sequences of atoms are established. These stacking sequences of atoms are separated into two models on the basis of the alternative chalcogen atoms in the MSSe (M = Mo, W) monolayers.^34^ Model-I represents the SiC-SMSe heterostructures, whereas model-II represents the SiC-SeMS heterostructures. The stacking sequence of atoms in model-I is: (a) Si atom is on the hollow side, while C atom is arranged on top of the Mo(W) atoms; (b) Si atoms are placed on top of the S(Se) atoms, while C is placed on the top of Mo(W); (c) C atoms are placed on top of the S(Se) atoms, while Si is placed on the top of Mo(W); (d) C atom is on the hollow side, while Si atom is arranged on top of the Mo(W) atoms for stacking; (e) C atom is on the hollow side, while Si atom is arranged on top of the S(Se) atoms, for stacking; (f) Si atom is on the hollow side, while C atom is arranged on top of the Se(Se) atoms. We have also relaxed all the similar stacking sequences of atoms in model-II as discussed. Six stacking configurations of model-I SiC-MSSe heterostructures are illustrated in Fig. 1.

**Fig. 2:**
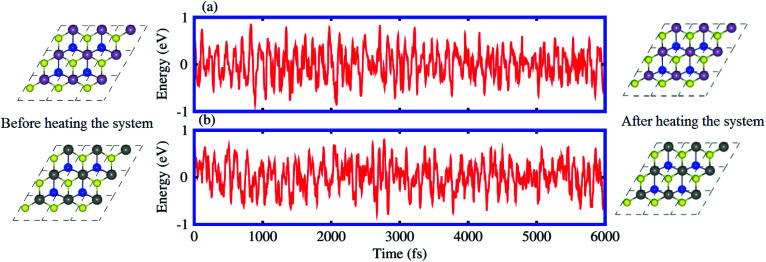
Model-I, *ab initio* molecular dynamics calculations of the thermal stability of (a) SiC-MoSSe and (b) SiC-WSSe vdW heterostructures.

The binding energy (*E*_b_) of heterostructures is also calculated to check the structural stability as follows: *E*_b_ = *E*_SiC-MSSe_ − *E*_SiC_ − *E*_MSSe_. The binding energy values of SiC-MSSe heterostructures for different stacking configurations are listed in Table 1. One can find from Table 1 that the (*f*) stacking configuration of SiC-MSSe for both the model-I and model-II heterostructures has the shortest interlayer distance and the lowest binding energy. Thus, the stacking (*f*) for both models is the energetically favorable stacking configuration. Therefore, we next focus only on the (*f*) stacking configuration of SiC-MSSe (M = Mo, W) heterostructures. The AIMD simulations of the SiC-MSSe heterostructures at room temperature are depicted in Fig. 2. One can observe that there are no geometric reconstructions or bonds broken after heating the system at 300 K for 6 ps, confirming that these systems are thermally stable even at room temperature.

**Table 1:** Lattice constant (*a*), binding energies (*E*_b_), interlayer distances (*d*), band gap (*E*_g_ in eV), and valence and conduction band edges (*E*_VB_, *E*_CB_ in eV) for all heterostructures

Heterostructures	Model-I		Model-II	
	SiC-MoSSe	SiC-WSSe	SiC-MoSSe	SiC-WSSe
*a* (Å)	3.17	3.18	3.18	3.18
*E*_(a)_ (eV)	−0.03179	−0.02775	−0.01088	−0.02379
*d* (Å)	3.0	3.21	3.42	3.17
*E*_(b)_ (eV)	−0.03194	−0.02794	−0.02281	−0.02624
*d* (Å)	2.94	3.23	3.39	3.31
*E*_(c)_ (eV)	−0.03102	−0.02795	−0.02278	−0.02624
*d* (Å)	2.92	3.34	3.44	3.08
*E*_(d)_ (eV)	−0.03124	−0.03124	−0.01572	−0.02031
*d* (Å)	3.12	3.12	3.41	3.39
*E*_(e)_ (eV)	−0.03105	−0.03182	−0.02374	−0.01405
*d* (Å)	3.20	3.16	3.38	3.47
*E*_(f)_ (eV)	−0.03208	−0.03370	−0.02375	−0.02778
*d* (Å)	2.90	2.83	3.00	2.95
*E*_g_ (PBE)	0.612	1.422	1.12	1.483
*E*_g_ (PBE + SOC)	0.514	1.30	1.08	1.33
*E*_g_ (HSE06)	1.74	1.88	2.16	2.34
*E*_VB_ (HSE06)	1.722	1.792	2.020	2.111
*E*_CB_ (HSE06)	−0.0185	−0.0885	−0.1400	−0.2305
*E*_VB_ (PBE)	1.208	1.65	1.411	1.68
*E*_CB_ (PBE)	0.494	0.228	0.291	0.198
*E*_VB_ (PBE + SOC)	1.157	1.587	1.393	1.604
*E*_CB_ (PBE + SOC)	0.545	0.291	0.309	0.274

**Fig. 3:**
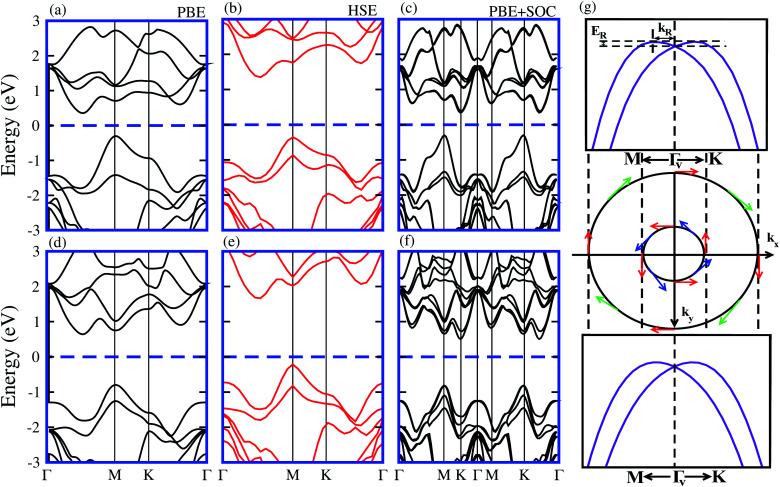
Band structures in model-I of SiC-MoSSe (a, b and c) and SiC-WSSe (d, e and f) vdW heterostructures using PBE, HSE06 and PBE + SOC functionals, respectively. (g) Schematic of spin texture around Γ_v_, and magnified view of valence and conduction band-splitting at the K-point and Rashba spin splitting around Γ_v_.

It is well known that the PBE functional underestimates the band gap of materials, while the hybrid functional HSE06 method can be used to obtain a more accurate band gap as compared to the experimental measurements.^35,36^ The electronic band structures of model-I of the SiC-MSSe heterostructures obtained from PBE, HSE and PBE + SOC approaches are depicted in Fig. 3. One can find that the SiC-MSSe heterostructures for model-I show indirect band gap semiconductors. The PBE, HSE06 and PBE + SOC methods show the same trends in the band structures of the SiC-MSSe heterostructures. HSE06 predicts the largest band gap, whereas PBE + SOC exhibits the smallest band gap due to the existence of band splitting. The band gap values of SiC-MSSe heterostructures for both models are listed in Table 1. We can find that the band gap of model-II is larger than that of model-I and the band gap of the SiC-WSSe heterostructure is larger than that of the SiC-MoSSe heterostructure. Energetically degenerate valleys at the valence and conduction band edges give the systems under study potential for valleytronics.^21,37^ Although larger spin splitting may be observed in the case of SOC included in the HSE06 functional than in the PBE functional,^6^ it accounts for the absence of the Rashba effect at the Γ_v_-point of BZ.^21^ Furthermore, it is clear that PBE is the local exchange-correlation functional, while HSE06 addresses the influence of the nonlocal exchange correlation functional.^38,39^ Therefore, the choice of exchange-correlation functional strongly affects the size of SOC splitting.^40^ Obviously, larger (less) spin splitting (Rashba splitting) is observed at the HSE06-SOC level than for PBE-SOC.^21^ Therefore, we mainly focus on PBE-SOC calculations here. Spin polarization of two valence bands at the Γ_v_-point satisfies *σ*(−*k*) = *σ*(*k*), while spin arrows in a clockwise pattern in the zone center are responsible for Rashba spin splitting, as plotted in the spin texture in Fig. 3(g). Rashba spin splitting can be defined by *α*^*K*,*M*^_R_ = 2*E*^*K*,*M*^_R_/*k*^*K*,*M*^_R_, where *α*^*K*,*M*^_R_ represents the Rashba parameter, *E*^*K*,*M*^_R_ represents the Rashba energy, *k*^*K*,*M*^_R_ represents the momentum offset along the Γ − *K* and Γ − *M* directions.^41^ The *α*^*M*^_R_ presented in Table 2 is slightly different from *α*^*K*^_R_, indicating that these parameters are not sensitive to the choice of directions in BZ for SiC-MSSe vdW heterostructures. Hence, considerable Rashba splitting with energy level splitting due to spin orbit interaction makes SiC-MSSe (M = Mo, W) vdW heterostructures promising candidates in spintronics and valleytronics.^21^

**Table 2:** Rashba energy (*E*^*M*^_R_ and *E*^*K*^_R_ in meV), momentum offset (*K*^*M*^_R_ and *K*^*K*^_R_ in Å) and Rashba parameter (*α*^*M*^_R_ and *α*^*K*^_R_ in eV Å) along the *K* and *M* directions for all heterostructures

Heterostructures	Model-I		Model-II	
	SiC-MoSSe	SiC-WSSe	SiC-MoSSe	SiC-WSSe
*E* ^*M*^_R_	0.0099	0.0059	0.0029	0.0048
*E* ^*K*^_R_	0.0101	0.0051	0.0029	0.0049
*K* ^*M*^_R_	0.0980	0.0630	0.0313	0.0317
*K* ^*K*^_R_	0.0850	0.0697	0.0313	0.0328
*α* ^*M*^_R_	0.2020	0.1869	0.1877	0.3049
*α* ^*K*^_R_	0.2365	0.1449	0.1876	0.3033

**Fig. 4:**
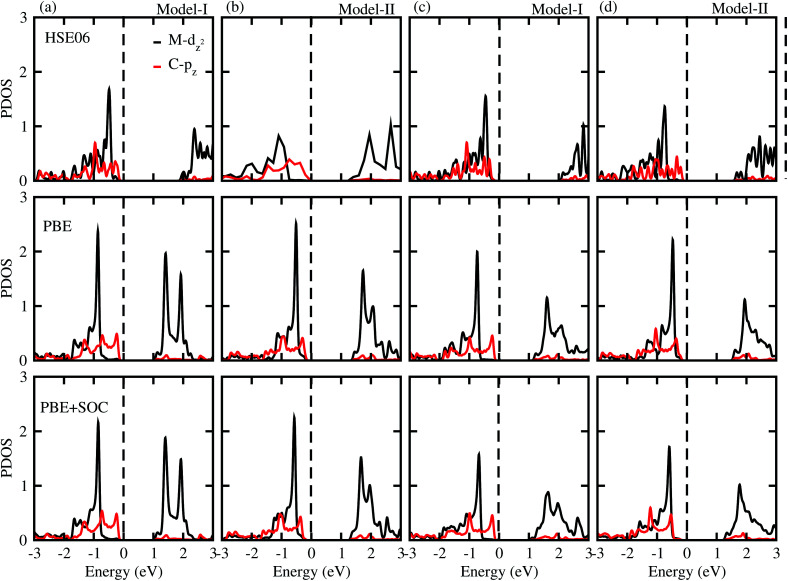
(a) Partial density of states using HSE06 (row I), PBE (row II), and PSE-SOC (row III) functionals of SiC-MoSSe in (a) model-I (b) model-II and of SiC-WSSe in (c) model-I and (d) model-II vdW heterostructures.

**Fig. 5:**
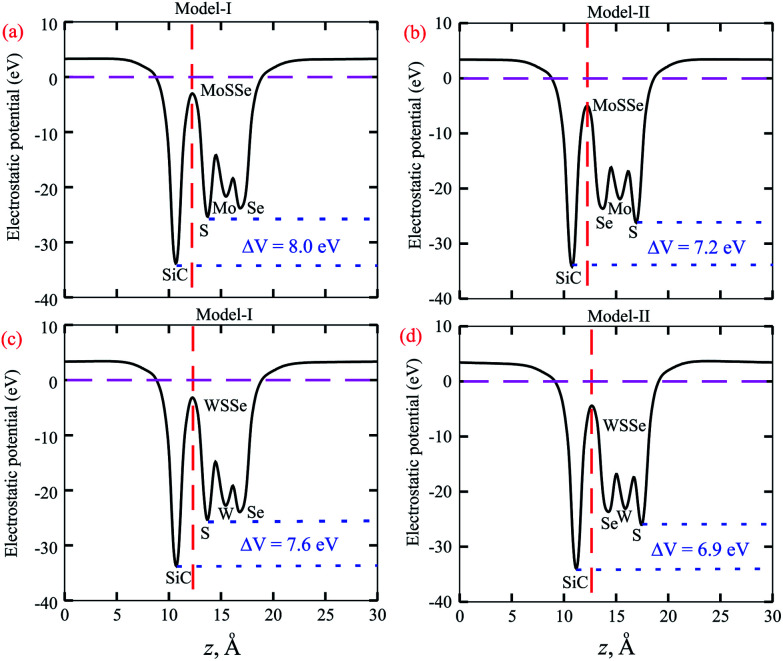
Average electrostatic potentials of (a) SiC-MoSSe, (c) SiC-WSSe for model-I and (b) SiC-MoSSe, (d) SiC-WSSe for model-II. Δ*V* represents the potential drop across the heterostructure. The dashed red line represents the heterostructure interface.

To gain more insight into the band alignment of the SiC-MSSe heterostructures, we further calculate the partial density of states (PDOS), as depicted in Fig. 4. We can see that the VBM and CBM of SiC-MSSe (M = Mo, W) vdW heterostructures in both model-I and model-II originate from different layers (Mo(W) – d_*z*^2^_, C – p), hence showing the type-II band alignment, which physically splits up the pair of positive and negative charges in different layers.^42^ Strong bonding between Mo(W) d_*xy*_, d_*yz*_ and d_*xz*_ with S(Se) p orbitals generates significant splitting at bonding and anti-bonding states. Therefore, Mo(W) d_*xy*_, d_*yz*_ and d_*xz*_ orbitals do not participate at the band edges. An external electric field is essential for net charge separation inside the same bilayer system to create type-II band alignment.^43^ The charge density difference shows that the majority of charge moves from C atoms to S atoms at the interface of SiC-MSSe (M = Mo, W) vdW heterostructures and charge is also driven from the SiC layer to the MSSe (M = Mo, W) layer in these heterostructures. The SiC monolayer becomes p-doped, while the Janus monolayer becomes n-doped after making the heterostructures. The small charge transfer in these layers shows weak interactions between the materials, as depicted in Fig. 5.

The calculated plane-average electrostatic potential of SiC-MSSe heterostructures is depicted in Fig. 5. One can observe that SiC layer has a deeper potential than the MoSSe and WSSe monolayers, indicating that electrons are moved from the MoSSe and WSSe monolayers to the SiC monolayer. Moreover, model-I represents the SiC-SMSe stacking configuration, whereas model-II represents the SiC-SeMS stacking configuration of the SiC-MSSe heterostructures. Due to the difference in electronegativity between S (2.58) and Se (2.55), the potential of the S layer is deeper than that of the Se layer, as depicted in Fig. 5. The potential drops between the SiC and MoSSe (WSSe) layers are 8.0 eV (7.2 eV) and 7.6 eV (6.9 eV) for model-I (model-II), respectively. Therefore, the excitonic behaviour of single layers of SiC and Janus monolayers can be different from that of the heterostructures, which help them to facilitate the separation of electrons and holes.^44^ The calculated work function along the *z* direction is 2.0 eV (1.7 eV) and 1.8 eV (1.9 eV) for SiC-MoSSe and SiC-WSSe in model-I (model-II), respectively. In the case of the monolayers, it has been shown that the Janus monolayers have higher work function than the SiC monolayer; hence the former (latter) will have positive charge (SiC) due to the high electrostatic induction, which enhances the power conversion efficiency.^34,45^

**Fig. 6:**
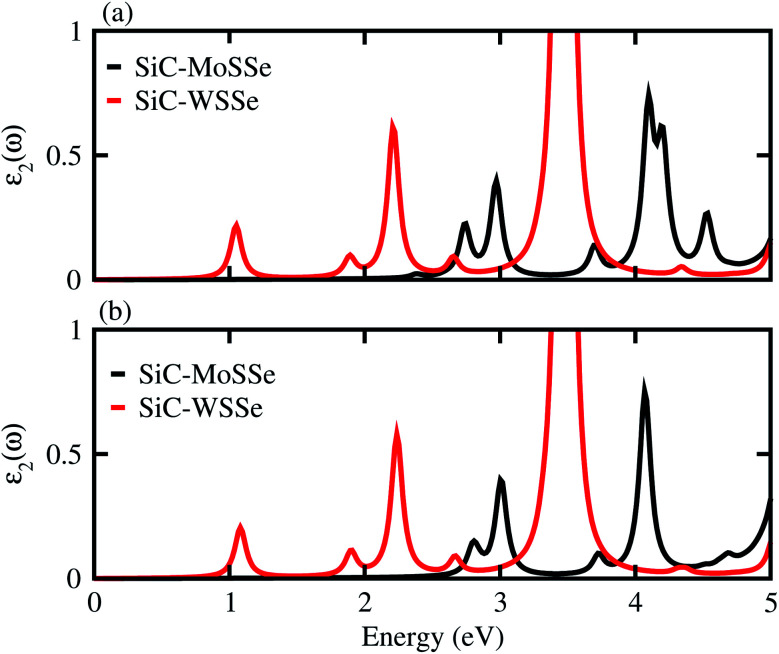
Imaginary part of the dielectric function of SiC-MSSe (M = Mo, W) in (a) model-I (b) model-II vdW heterostructures.

**Fig. 7:**
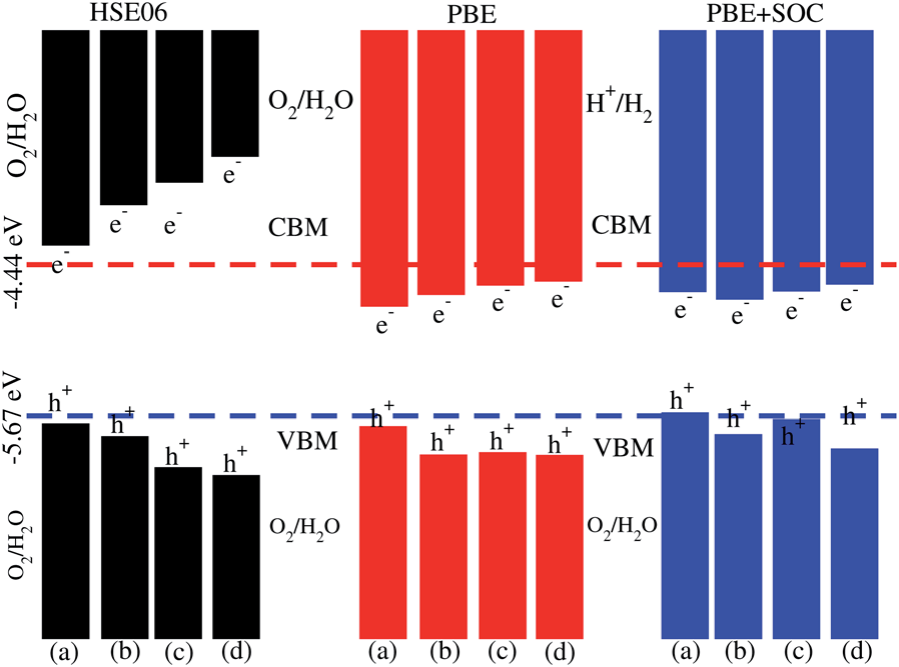
Valence and conduction band edge alignment of SiC-MoSSe (a) model-I, (b) model-II and SiC-WSSe (c) model-I, (d) model-II vdW heterostructures with standard oxidation (−5.67 eV) and reduction (−4.44 eV) potentials for water splitting.

The dielectric function provides a strong connection between experimental measurement and theoretical prediction related to excited-state characteristics and plays a key role in the characterization of a novel class of materials for advanced technological device applications. The observed first excitonic peaks (binding energies) appear at 2.65 (0.91) eV and 1.2 (0.74) eV for model-I, and 2.9 (0.68) eV and 1.3 (1.09) eV for model-II of the SiC-MoSSe and SiC-WSSe heterostructures, respectively. A red shift can also be observed in the position of the excitonic peaks from SiC-MoSSe to SiC-WSSe, while a blue shift is seen from model-I to model-II, as depicted in Fig. 6. Absorption spectra in the visible region of the spectrum followed by several peaks in the ultraviolet region for both models make SiC-MSSe (M = Mo, W) vdW heterostructures suitable for photovoltaic applications. Semiconductors with a suitable band gap can efficiently utilize solar energy to dissociate water and generate hydrogen.^46,47^ Thus, photocatalytic water splitting can be used for clean renewable energy.^48,49^ In the photocatalytic process, the electrons (holes) reduce (oxidize) water.^50^ For this process, the oxidation (reduction) potential of 0 (1.23) eV must be less (more) than the conduction (valence) band.^51^ This means that the semiconductor band gap must be greater than 1.23 eV (Fig. 7).

Photocatalytic water splitting for the SiC-MSSe (M = Mo, W) vdW heterostructures is investigated by using Mulliken electronegativity: *E*_VBM_ = *χ* − *E*_elec_ + 0.5*E*_g_ and *E*_CBM_ = *E*_VBM_ − *E*_g_.^52,53^ It is clear that the standard oxidation and reduction potentials on the hydrogen scale for photocatalytic water splitting are −4.44 eV and −5.67 eV, respectively.^54,55^ Thus, in order to obtain the band edge positions of the CB and VB with respect to standard oxidation on the hydrogen scale, the Fermi level is set to be −4.44 eV.^21^ The band edge potentials of SiC–Janus heterostructures in aqueous solutions with reduction and oxidation potential using HSE06, PBE and PBE + SOC functionals are given in Table 1. The VB and CB are set to 1.23 eV and 0 eV, which are equal to −5.67 eV and −4.44 eV at pH = 0.^52^ For SiC-MSSe (M = Mo, W) vdW heterostructures, both the VB and CB potentials calculated by the HSE06 functional straddle the standard redox band edges, satisfying the requirements for water splitting at pH = 0. The band edge potentials using PBE and PBE + SOC calculations show that SiC-MSSe vdW heterostructures are more positive than the required VB potential, showing good responses for the oxidation of water. A similar trend is also demonstrated for SiC-TMDCs, JTMDC-JTMDCs and GeC-MSSe heterostructures.^20,21,34^ Hence, we conclude that the SiC-MSSe heterostructures can be considered as promising candidates for the large scale production of solar hydrogen.

## 4 Conclusion

Using DFT calculations, we have investigated the electronic structure, Rashba effect, optical and photocatalytic performance of SiC-MSSe (M = Mo, W) vdW heterostructures. The favorable stacking patterns of two models with alternative chalcogen atoms in SiC-MSSe vdW heterostructures are also dynamically and energetically stable. SiC-MoSSe shows type-II(-I) band alignment for model-I(-II), respectively, while SiC-WSSe remains type-II in both models of the heterostructure. The SOC effect induces considerable Rashba spin splitting in both models of SiC-MSSe (M = Mo, W) vdW heterostructures, providing a platform for understanding the design of spintronic devices. The calculated plane-average electrostatic potentials show that SiC has deeper potential than the MoSSe and WSSe monolayers, while the calculated work functions along the *z* direction are 2.0 eV (1.7 eV) and 1.8 eV (1.9 eV) for SiC-MoSSe and SiC-WSSe in model-I (model-II), respectively. The imaginary part of the dielectric function of SiC-MSSe (M = Mo, W) vdW heterostructures reveals that the first optical transition is due to the bound excitons, and it possesses high absorption in the visible region. Appropriate band alignments for both models with the standard water redox potentials allow them to dissociate water into H^+^/H_2_ and O_2_/H_2_O.

## Conflicts of interest

There are no conflicts to declare.

## Supplementary Material
